# On the role of L-type Ca^2+^ and BK channels in a biophysical model of cartwheel interneurons

**DOI:** 10.1371/journal.pcbi.1013382

**Published:** 2026-04-15

**Authors:** Matteo Martin, Jonathan E. Rubin, Morten Gram Pedersen

**Affiliations:** 1 Department of Information Engineering, University of Padova, Padova, Italy; 2 Department of Mathematics, University of Pittsburgh, Pittsburgh, Pennsylvania, United States of America; 3 Padova Neuroscience Center, University of Padova, Padova, Italy; University of Groningen: Rijksuniversiteit Groningen, NETHERLANDS, KINGDOM OF THE

## Abstract

Cartwheel interneurons (CWCs) in the auditory system, which contribute to auditory processing and pathologies, exhibit a range of activity patterns, including bursting, spiking, and complex spiking. Although experiments have shown how these patterns can vary across individual neurons, the field has lacked a computational framework in which to explore the contributions of particular currents to these observations and to generate new predictions about the effects of pharmacological manipulations. We present a conductance-based CWC computational model, which captures the diversity of CWC activity patterns observed experimentally and suggests parameter changes that may underlie differences across cells. Specifically, we show using direct simulations and bifurcation diagrams that one parameter tuning yields a regular spiking phenotype in which the onset of activity, as input current is increased, takes the form of regular spiking and other tuning that gives a complex spiking phenotype in which bursting occurs at the spike onset and regular spiking only occurs over a narrow input range before it gives way to complex spiking. We next investigate the effects of the BK-type potassium current blocker iberiotoxin and the L-type calcium current blocker nifedipine. Our model reproduces the transitions to complex spiking and regular spiking, respectively, observed experimentally when these drugs are administered. In addition to the full model, we present a reduced model that preserves CWC dynamic regimes. We classify the reduced model variables in terms of distinct dynamic timescales and show that the key transitions in dynamic patterns under administration of iberiotoxin and nifedipine can be explained based on equilibria of the averaged dynamics of the slowest model variables, in a regime where the faster model variables exhibit oscillations. Overall, this study predicts how changes in parameters will influence CWC behavior, suggests how bifurcations contribute to changes in CWC dynamics, and provides a theoretical foundation that supports our simulation findings.

## Author summary

Cartwheel interneurons (CWCs) are the most common class of inhibitory interneurons in an auditory brainstem region involved in sound localization and are believed to be important for auditory processing and pathologies. Distinct patterns of CWC activity have been observed experimentally in a variety of conditions. In this work, we present two novel computational models that simulate the factors contributing to CWC dynamics. By harnessing this framework, we are able to reveal the contributions of key ion currents to modulating CWC activity. Indeed, we find that the factors present in CWC neurons can produce a complicated dynamic landscape, with a wide range of output patterns possible as the relative strengths of these factors are varied. Overall, our models represent useful tools for understanding experimental results and generating new predictions about CWC behavior. In particular, in the more reduced of the two models, we can perform mathematical analysis to make more detailed predictions about the effects of current modulation on whether CWC neurons will exhibit regular spiking or more complex forms of outputs.

## Introduction

Cartwheel cells (CWC) are cross-species [1–6], glycinergic interneurons located in the dorsal cochlear nucleus (DCN). As the predominant form of inhibitory neuron in the DCN [7], they regulate the electrical activity of fusiform neurons [8] and thus they can play a role in auditory processing as well as in pathologies such as tinnitus [9–11]. To understand auditory function and dysfunction, it is therefore crucial to study the mechanisms driving and modulating CWC electrical activity.

CWCs exhibit diverse activity patterns classified as spiking, bursting, and complex spiking [12]. Complex spikes include both large amplitude action potentials (LAOs) and smaller amplitude oscillations (SAOs), which Kim et al. call spikelets [12]. While certain forms of bursting can also feature SAOs, complex spiking differs from neuronal bursting [13–15] in that it lacks the prolonged epochs of quiescence between groups of spikes that occur in burst patterns. Functionally speaking, complex spiking may play a crucial role by influencing the susceptibility of neighboring neurons to Ca^2+^-dependent plasticity [16]. Experimental studies have distinguished CWCs that are spikers from complex spikers based on the oscillation patterns that they display at the onset of activity as an applied current is varied. Both CWC types, however, can exhibit forms of complex spiking in response to sufficient increases in depolarizing applied current. Moreover, treatment with nifedipine (an inhibitor of L-type Ca^2+^ channels) can upregulate the likelihood of activating regular spiking, while treatment with iberiotoxin (an inhibitor of big-conductance, Ca^2+^-dependent K^+^ (BK) channels) can downregulate the occurrence of regular spiking, in both classes of CWCs [12,17].

In this work, we introduce a novel eleven-dimensional, conductance-based ordinary differential equation (ODE) model [18] for CWCs, which we use to analyze the dynamic mechanisms underlying these observations about CWC dynamics. Specifically, we first use one-parameter bifurcation analysis to show how alterations in the balance of calcium and potassium currents in CWCs can lead to the observed differences in spiker and complex spiker dynamics at activity onset. We next show that the model can reproduce experimental results where BK or L-type channels were blocked by iberiotoxin or nifedipine, respectively, and we then explore effects of more modest reductions, as well as increases, of BK and L-type Ca^2+^ conductances. With a model, we can consider both the pharmacological lesions performed experimentally as well as more subtle changes in channel activity, such as the effects of lower drug doses [19,20], neuromodulation [21], or gain-/loss-of-function mutations, transcription or trafficking defects, and other consequences of channelopathies [22]. To this end, we create heatmaps (cf. [23]) illustrating how a morphological index, defined as an extension of the firing number [24], changes when the applied current (*I*_*App*_) and the BK or L-type Ca^2+^ conductance (*g*_*BK*_ or *g*_*CaL*_) are varied, and integrate these results with two-parameter continuation of the most important bifurcations.

We further investigate model responses to simulated variations in BK and L-type conductances by introducing a model reduction and applying averaging theory [25,26]. The results of this investigation demonstrate a relation between the spiking patterns observed under parameter variation in simulations and the stability properties of an equilibrium point of a superslow component of the model system. Overall, our models can be used to predict how parameter changes will affect CWC behavior and to identify ways to alter CWC dynamics, and they provide a theoretical underpinning that substantiates our simulation results.

## Results

### Two classes of CWC conductance-based model dynamics

We developed a novel conductance-based CWC interneuron model, based on a variety of experimental observations about these cells (see *Methods*, equations (1)). We used our model to showcase that the experimentally observed differences between spiker and complex spiker CWCs do not necessarily correspond to the expression of different sets of ion channels, which would be reflected in a different model structure, but can arise from minor changes in ion channel whole-cell conductances. The two classes of CWCs are defined based on the voltage dynamics that they exhibit at the onset of activity as an applied current, *I*_*App*_, is increased from hyperpolarizing levels. Specifically, the parameter set capturing the behaviour of a spiker CWC compared to the complex spiker has lower whole-cell conductances of voltage-gated K^+^ (*g*_*KV*_) and L-type Ca^2+^ (*g*_*CaL*_) channels, but higher persistent Na^+^ (*g*_*NaP*_) and Ca^2+^-dependent K^+^ (*g*_*KCa*_) conductances.

**Fig 1:**
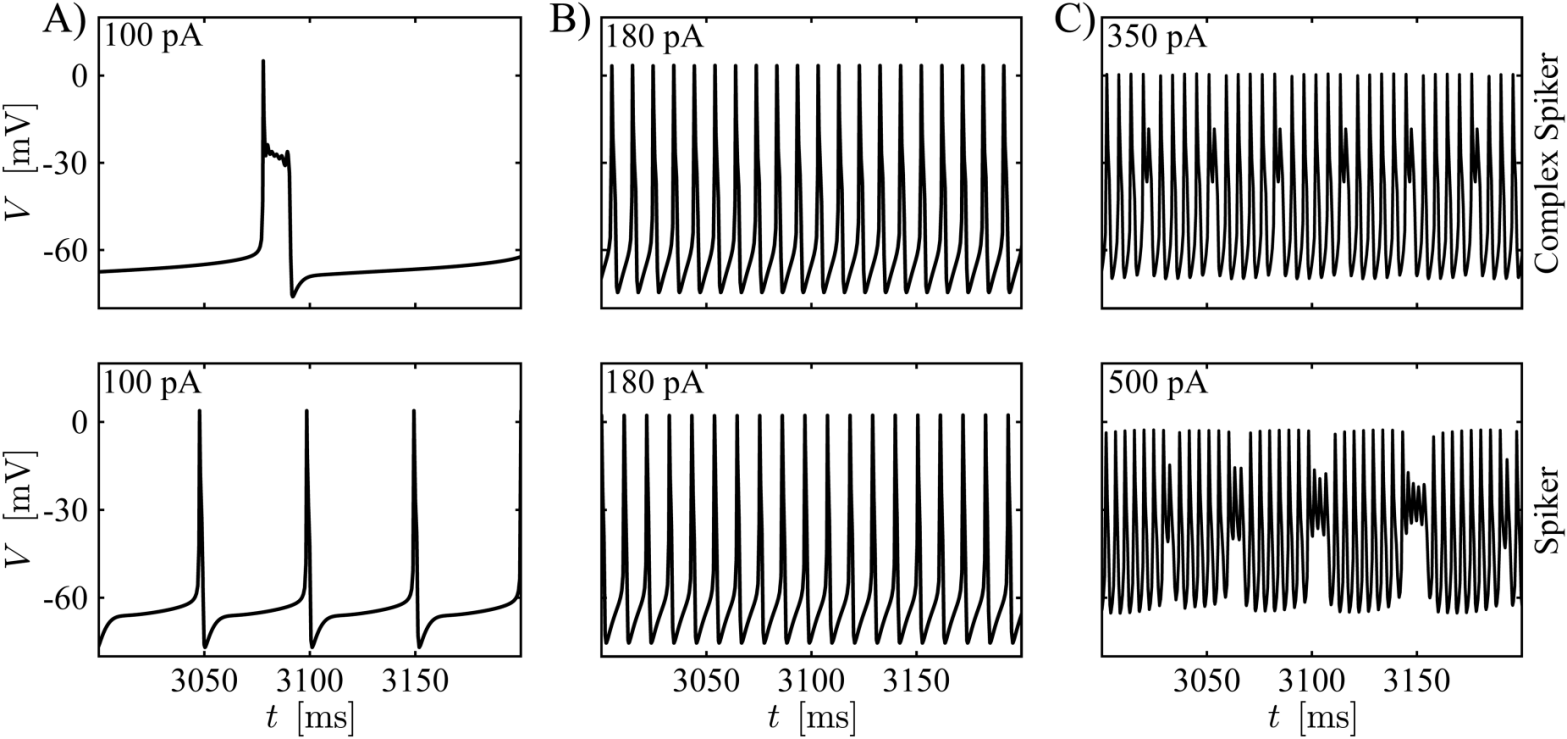
CWC interneuron model time series. **(A)** At low *I*_*App*_, the complex spiker model exhibits pseudo-plateau bursting (top), whereas the spiker model displays spiking (bottom). **(B)** Both the complex spiker (top) and the spiker models show regular spiking for larger *I*_*App*_. **(C)** Both models produce complex spiking for still greater *I*_*App*_, although larger values are needed for the spiker (bottom) than for the complex spiker. In all panels, the *I*_*App*_ value used appears in the upper left.

For these combinations of parameters, the two classes of cells qualitatively capture the progressions of activity patterns observed experimentally as *I*_*App*_ is increased [12,27]. For low values of *I*_*App*_, complex spikers and spikers exhibit pseudo-plateau bursting and regular spiking, respectively, as shown in Fig 1A, in agreement with the experiments for low levels of stimulating current in Fig 5A (respectively, Fig 5B) in [27] and Fig 5Ai (respectively, Fig 5Aii) in [12]. For the traces in Fig 1A, the firing frequencies are ∼6 Hz and ∼20 Hz for bursting and spiking, respectively, which are comparable to those presented in [12] (8 Hz and 28 Hz, respectively) but slower than those presented in [27] (17 Hz and 66 Hz, respectively).

For larger *I*_*App*_, as observed in experiments (e.g., Fig 2A in [12] for similar applied current values), the two types of interneurons both engage in regular spiking (Fig 1B). Also in this case, the model exhibits firing frequencies (95 Hz and 85 Hz for the complex spiker and spiker, respectively) that are close to those associated with the previously mentioned experiments (64 Hz and 90 Hz, respectively). Finally, for sufficiently high *I*_*App*_, the interneurons generate complex spiking activity (Fig 1C), as in Fig 10A in [12]. The experimental firing frequency (130 Hz) is comparable with the firing frequency in the model simulations shown in Fig 1C (160 Hz and 180 Hz for the complex spiker and the spiker, respectively).

**Fig 2:**
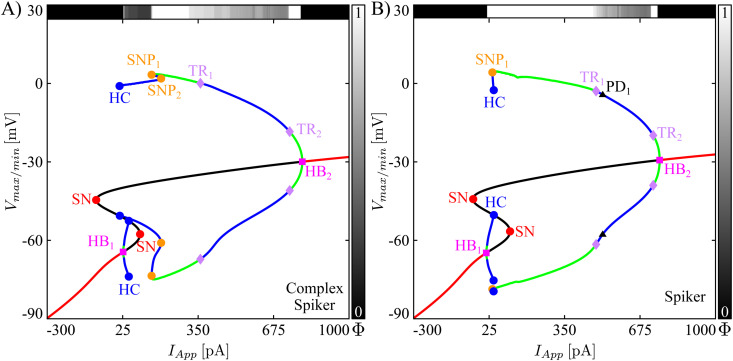
Bifurcation diagrams for the CWC model. Bifurcation diagrams with respect to *I*_*App*_ for two parameter sets corresponding to complex spikers **(A)** and spikers **(B)**. Labeled geometric symbols denote bifurcations: The orange (red) dots are associated with saddle-node bifurcations of periodic orbits (SNP) (of critical points (SN)), the magenta squares with Andronov-Hopf bifurcations (HB), the blue dots with distinct homoclinic bifurcations (HC) associated with HB_1_ and HB_2_, the violet diamonds with torus bifurcations (TR). The red/black continuous curve indicates *V* values for stable/unstable critical points. The green/blue curves represent the maximum and the minimum voltages along stable/unstable periodic orbits. The horizontal bar along the top of the BD shows how the firing number Φ changes along the system’s attracting solution with variations in *I*_*App*_. The greyscale coding used for this bar is indicated on the right (white: Φ=1; black: Φ=0). In particular, white intervals in the top bar correspond to regular spiking regimes.

To characterize the model dynamics and the difference between the two versions of the model more thoroughly, we computed bifurcation diagrams (BD) for both the complex spiker and spiker models, with *I*_*App*_ as the bifurcation parameter (Figs 2A and 2B). Each bifurcation diagam in Fig 2 shows a variety of labeled bifurcation events. We denote the *I*_*App*_ value where a bifurcation B occurs by *I*_*App*,B_, where B can denote any bifurcation type. Along with each *I*_*App*_-BD, we include a bar above the main plot showing how the firing number Φ [24] changes with *I*_*App*_. For each *I*_*App*_, this index is defined as Φ=L/(L+s) where *L* is the number of large-amplitude oscillations and *s* is the number of small-amplitude oscillations occurring per oscillation cycle within the system’s stable oscillation pattern approached by the trajectory with initial conditions taken as the stable equilibrium point present for *I*_*App*_ = 0 pA. The firing number takes the value 1 for regular, large amplitude spiking, decreases as small amplitude oscillations become more prevalent, and is equal to 0 for tunings in which the neuron is silent. More details are provided in the Firing number section of *Methods*. The bars on the right of the *I*_*App*_-BDs show the mapping between shades of grey and values of Φ. In particular, black and white correspond to a zero and unitary Φ, respectively. For values of *I*_*App*_ where the model shows bistability, for example just to the left of TR_1_ in Fig 2A, the firing index reflects the system’s long-term behavior with the given initial conditions.

The branch of equilibria in each of the BDs in Fig 2 has the typical S shape with folds at saddle-node (SN) bifurcations, as seen for many models of cellular transmembrane electrical activity. We observe that both model neurons are silent if a hyperpolarizing (negative) current is applied. This situation persists as current is increased through zero until it becomes depolarizing and reaches $I_{App,HB_{1}}$, which is equal to 27 (12) pA in the complex spiker (spiker) model, where a supercritical Andronov-Hopf bifurcation (HB_1_) occurs. At this critical value, the stable equilibrium point loses its stability, and the two models start to behave differently. In both cases, although HB_1_ is supercritical and hence gives rise to a family of periodic solutions that are initially stable, these solutions destabilize almost immediately and remain unstable throughout the remainder of their extent, up through termination in a homoclinic bifurcation (HC). Additional features in the BD for the complex spiker include two saddle node bifurcations of periodic orbits (SNP_1_, SNP_2_). These are linked by a family of unstable periodic orbits (POs); specifically, SNP_2_ is a coming together of two unstable PO families, while SNP_1_ involves the unstable family along with a family of stable POs (green in Fig 2A).

Note that for the complex spiker, over a significant interval of *I*_*App*_ values above $I_{App,HB_{1}}$, no stable solution is evident in the bifurcation diagram (Fig 2A). Here, the complex spiker exhibits a form of activity that combines LAOs and SAOs (Fig 1A), known as pseudo-plateau bursting, with small Φ. With additional increases in *I*_*App*_, at the saddle-node of periodic orbits bifurcation (SNP_1_), the complex spiker switches to regular spiking with Φ=1 (green curves between SNP_1_ and TR_1_, Fig 2A). For the spiker (Fig 2B), on the other hand, at an *I*_*App*_ value just slightly above $I_{App,HB_{1}}$, a saddle-node of periodic orbits bifurcation (SNP_1_) occurs, which gives rise to a stable family of large amplitude, periodic spiking solutions (green curves between SNP_1_ and the torus bifurcation TR_1_). Hence, the spiker exhibits an almost-immediate transition from quiescence to regular spiking, with Φ=1.

Overall, the key features that distinguish the complex spiker from the spiker in this model are: the added complexity of the leftmost part of the branch of periodic orbits in the complex spiker BD for *I*_*App*_ between $I_{App,HB_{1}}$ and $I_{App,SNP_{1}}$, the larger value of $I_{App,SNP_{1}}\approx 150$ pA where the onset of regular spiking occurs for the complex spiker, and the significantly wider interval of *I*_*App*_ values yielding regular spiking for the spiker case.

If we increase *I*_*App*_ far enough above $I_{App,SNP_{1}}$, both models’ dynamics switch to complex spiking. We notice that, contrary to the previous cases, where the transitions between different states were directly associated with bifurcations in the *I*_*App*_-BD, the transition from spiking to complex spiking is not. Specifically, the transition occurs before the critical value $I_{App,TR_{1}}$, which is equal to 359 and 485 pA in the complex spiker and spiker models, respectively. Indeed, for the complex spiker, as *I*_*App*_ increases from $I_{App,SNP_{1}}$ toward $I_{App,TR_{1}}$, the firing number Φ starts to decrease before $I_{App,TR_{1}}$ is reached, reflecting the change in the signature of the model’s dynamics. Various forms of complex spiking activity persist in both models until the critical value of $I_{App,TR_{2}}$, which is equal to 744 and 732 pA in the complex spiker and spiker models, respectively, is reached. For *I*_*App*_ above $I_{App,TR_{2}}$, the models switch to stable, high-frequency but low-amplitude, regular spiking. Finally, both models enter a state of depolarization block with a stable equilibrium once *I*_*App*_ exceeds the Andoronov-Hopf bifurcation HB_2_ at $I_{App,HB_{2}}$, equal to 798 (759) pA in the complex spiker (spiker) model.

In summary, raising *g*_*KV*_ and *g*_*CaL*_ and lowering *g*_*NaP*_ and *g*_*KCa*_ (which is how the complex spiker phenotype is obtained in our model) alter spiker dynamics in two main ways. First, these changes interrupt the direct transition from quiescence to regular spiking via the introduction of a significant window of *I*_*App*_ values over which pseudo-plateau bursting occurs and an increase in the *I*_*App*_ value needed to achieve regular spiking. Second, they shrink the interval of *I*_*App*_ values over which regular spiking occurs, allowing complex spiking to kick in at relatively much lower *I*_*App*_.

### Inhibition of BK channels promotes complex spiking and bursting in CWCs

Iberiotoxin, a potent big-conductance K^+^ (BK) channel blocker, diminishes the likelihood of observing regular spiking in cartwheel interneurons and promotes the emergence of bursting or complex spiking phenomena [12,17]. Our model can reproduce these results and allows us to explore and predict effects of gradual reductions or increases in the BK channel conductance, *g*_*BK*_, across a range of *I*_*App*_ values.

**Fig 3:**
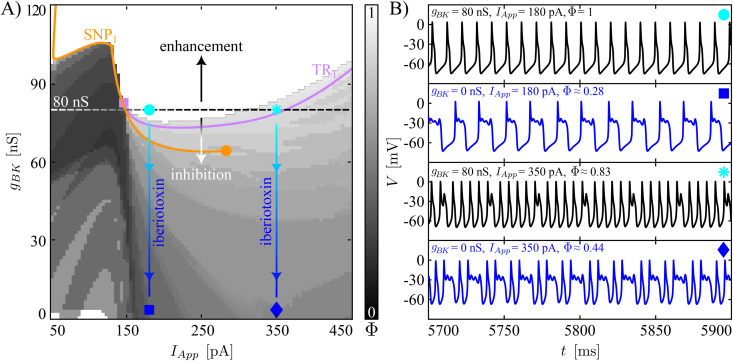
Responses of complex spikers to variation of *g*_*BK*_ and *I*_*App*_ and to iberiotoxin adminstration. **(A)** Two-parameter plane illustrating the heatmap for the index Φ, and the movement of SNP_1_ and TR_1_ with respect to parameters (*I*_*App*_, *g*_*BK*_). The vertical bar on the right of the plot corresponds to firing number Φ (cf. Fig 2). The horizontal black dashed line represents the baseline value of *g*_*BK*_ (80 nS) used in the model. The regular spiking (white) in this type of interneuron is highly sensitive to small reductions of *g*_*BK*_ because the baseline dynamics is set close to the border of the regular spiking domain. The vertical, blue lines represent effects of iberiotoxin administration. Each connects two symbols, the circle/asterisk and the square/diamond, respectively. The orange (purple) curve is the two-parameter continuation of the SNP_1_(TR_1_) shown in Fig 2. The purple square corresponds to the collision between the TR_1_ and the SNP_1_. The orange circle represents a cusp of SNP where SNP_1_ ends. **(B)** Model behavior at the parameter values indicated by the four markers presented in **(A)**. Blockade of *g*_*BK*_ converts regular spiking into bursting, while complex spiking persists with more SAOs relative to LAOs (smaller Φ).

**Fig 4:**
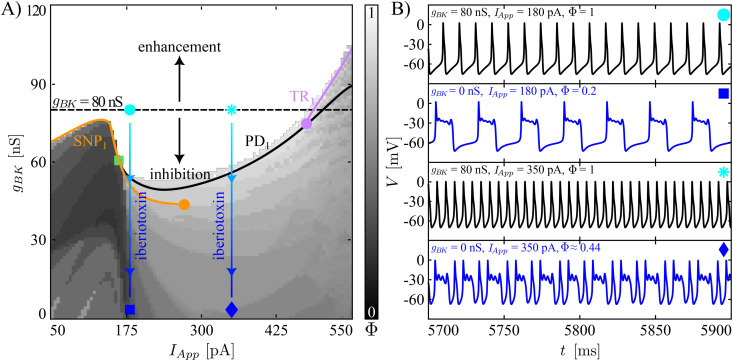
Responses of spikers to variation of *g*_*BK*_ and *I*_*App*_, and to iberiotoxin adminstration. Notation follows that of Fig 3. **(A)** Heatmap of the firing number Φ, and two-parameter curves of TR_1_, SNP_1_, and PD_1_ under variation of the two parameters *g*_*BK*_ and *I*_*App*_. The black curve shows the two-parameter continuation of the PD_1_ bifurcation, while the green square indicates the collision point of the PD_1_ with SNP_1_. Finally, the purple circle is a fold of TR. **(B)** Model dynamics at the different points in parameter space are indicated by markers of various shapes. Simulated iberiotoxin administration converts regular spiking to pseudo-plateau bursting (*I*_*App*_ = 180 pA) or complex spiking (*I*_*App*_ = 350 pA).

Figs 3A and 4A display results of systematic variation of *g*_*BK*_ and *I*_*App*_ for our two baseline model tunings in terms of heatmaps of the firing number Φ and the movement of those bifurcations illustrated in Fig 2 where the computed branch of limit cycles changes stability. In these figures, the baseline value of *g*_*BK*_ is indicated with a black, dashed horizontal line. Before exploring less extreme modifications of the BK conductance, we simulate the iberiotoxin experiments, assuming that the drug blocks all of the BK channels in the cell. The sample trajectories for the complex spiker presented in Fig 3B show that for *I*_*App*_ equal to 180 pA, the model under control conditions (*g*_*BK*_ = 80 nS) produces regular spiking, which is converted into pseudo-plateau bursting when iberiotoxin is administered (*g*_*BK*_ = 0 nS). The transition presented here matches the change in dynamics shown in Fig 7A in [12] from simple spiking to pseudo-plateau bursting when iberiotoxin was applied to a CWC, the type (complex spiker or spiker) of which was not specified. The number of SAOs in the experimentally obtained pseudo-plateau bursting was 1 or 2, whereas our model generates plateaus with 2 or 3 SAOs. For a stimulating current of 350 pA, the complex spiker produces complex spiking, which persists with a smaller Φ value under iberiotoxin administration (*g*_*BK*_ = 0 nS). Fig 4B shows the behavior of a spiker interneuron under control (*g*_*BK*_ = 80 nS) and iberiotoxin-treated (*g*_*BK*_ = 0 nS) conditions. For *I*_*App*_ equal to 180 pA or 350 pA, the spiker interneuron exhibits regular spiking, which is transformed into pseudo-plateau bursting or complex spiking, respectively, when iberiotoxin is administered, as experimentally observed (e.g., Fig 7 in [12]). Thus, both models exhibit similar responses to iberiotoxin, despite their differences in baseline dynamics.

Next, we consider more gradual reductions of *g*_*BK*_ (regions of the two-parameter plane below the black, dashed horizontal line). In the heatmaps (Figs 3A and 4A), the color-coding of Φ corresponds to that in Fig 2. In both cases, we see that starting from a regular spiking regime achieved by tuning of *I*_*App*_, reductions of *g*_*BK*_ cause decreases in Φ, representing a larger number of SAOs and/or a lower number of LAOs in the model’s periodic activity patterns. The same trend, with fewer LAOs and more SAOs as *g*_*BK*_ decreases, recurs for the complex spiker case when *I*_*App*_ is increased to 350 pA, corresponding to what is initially a complex spiking regime (Fig 3B). For the complex spiker, the region of regular spiking with *g*_*BK*_ equal to 80 nS is especially fragile, with small reductions in *g*_*BK*_ eliminating regular spiking (Fig 3A). This outcome arises because the complex spiker model, due to the chosen parameter settings, lies close to the border of the regular spiking domain. Another small window with Φ=1 emerges for the complex spiker with *I*_*App*_ small and *g*_*BK*_ close to 0 nS. However, unitary Φ values in this region do not correspond to regular spiking but rather to plateau-bursting with no SAOs during the active phase, i.e., very broad action potentials, each consisting of a spike and a depolarized plateau, separated by prolonged pauses. For the spiker case (Fig 4A), the regular spiking area around the *g*_*BK*_ baseline value (80 nS) is wider and more robust than that of the complex spiker. In this regime, a reduction of *g*_*BK*_ of at least 35% is needed to abolish regular spiking.

In contrast to the previous observations, when *g*_*BK*_ increases (regions of the two-parameter plane above the black, dashed horizontal lines), in both the complex spiker and the spiker, the range of *I*_*App*_ yielding regular spiking widens. In general, we note that although the switch between the complex spiker and spiker models involves changes in several conductances, the two models produce qualitatively similar heatmaps with respect to (*I*_*App*_, *g*_*BK*_), with a quantitative shift in the *g*_*BK*_ values associated with similar dynamics across the two models.

As observed in Fig 2, the stability of the limit cycle changes at SNP_1_ and TR_1_. In the complex spiker model, SNP_1_ predicts well the transition from bursting to spiking, whereas in both CWC phenotypes the location of TR_1_ approximately indicates the transition from simple spiking to complex spiking. The two-parameter continuation of these bifurcations are illustrated in Fig 3 and 4. The SNP_1_ curve terminates in a cusp of SNP (orange circle), where the SNP_1_ and the SNP_2_ meet. The continuation of TR_1_ for the complex spiker continues up to the collision with the SNP_1_ curve (purple square), whereas for the spiker interneuron it disappears as it coalesces with another torus bifurcation close to PD_1_ (purple circle). For this reason, in the spiker, we followed the PD_1_ bifurcation, which terminates in a collision with the SNP_1_ curve (green square). In both CWC phenotypes, as *g*_*BK*_ is reduced, the curve of SNP_1_ to the left of the purple (respectively, green) square marks the transition boundary between spiking and bursting in the complex spiker (respectively, spiker) interneuron. Instead, the TR_1_ (respectively, TR_1_ or PD_1_) curve approximately follows the complex spiking boundary in the complex spiker (respectively, spiker) interneuron. The fact that the heatmap does not correpond perfectly to the 2P-BD establishes that the bistability discussed above for the 1P-BD (Fig 2) persists when the maximal BK channel conductance is varied.

From the biological perspective, the BK channels activate along with P/Q-type Ca^2+^ currents and act on a depolarized transmembrane potential, providing a hyperpolarizing effect. When the BK conductance is set to 0 nS, one of the forces contributing to repolarization of the membrane potential towards the resting state is no longer present. For this reason, the membrane potential evolves around a depolarized plateau, where small fluctuations may occur. Both the regular spiking and the complex spiking cases (with few SAOs) start to show longer depolarized plateaus with a higher number of SAOs superimposed.

### Inhibition of the L-Type Ca^2+^ channel enhances regular spiking

The L-type Ca^2+^ channel current can be suppressed by the pharmacological agent nifedipine. Experimental application of this blocker abolished complex spiking activity in most CWC interneurons [12]. Our model reproduces this result and allows us to explore and predict the effects of varying degrees of reduction and increase of the L-type Ca^2+^ channel maximal conductance *g*_*CaL*_ across a range of *I*_*App*_ values.

**Fig 5:**
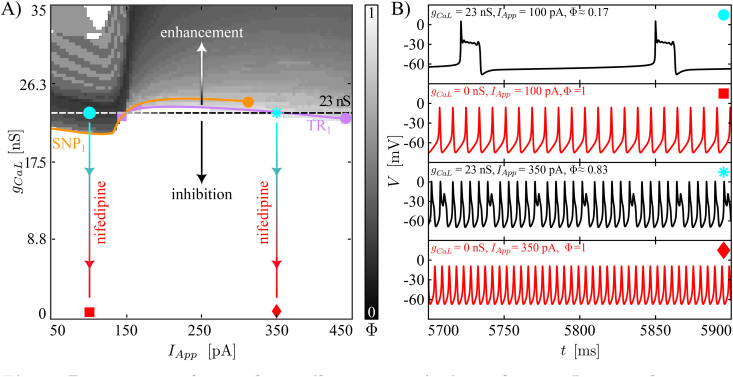
Responses of complex spikers to variation of *g*_*CaL*_, *I*_*App*_, and to nifedipine adminstration. The content of the current figure is organized as in Fig 3. **(A)** Heatmap showing how the firing number Φ changes, and the two-parameter continuations of TR_1_, SNP_1_, as *I*_*App*_ and *g*_*CaL*_ are varied. **(B)** Time series associated with the markers presented in panel **(A)**, corresponding to, from top to bottom, control conditions with relatively low *I*_*App*_, nifedipine administration with relatively low *I*_*App*_, control conditions with relatively high *I*_*App*_, and nifedipine administration with relatively high *I*_*App*_.

**Fig 6:**
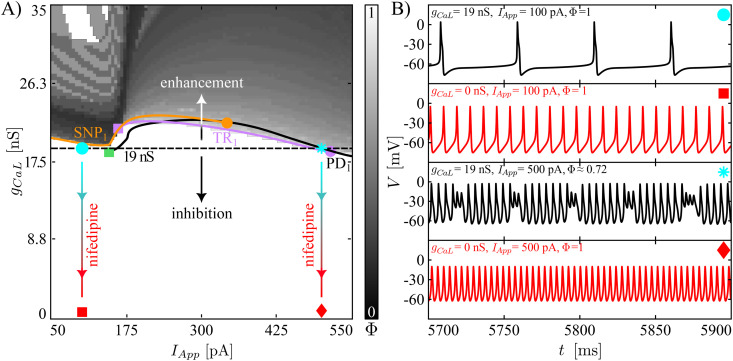
Responses of spikers to variation of *g*_*CaL*_, *I*_*App*_, and to nifedipine adminstration. The content of the current figure is organized as in Fig 3. **(A)** Heatmap with two-parameter bifurcation curves superimposed showing how the firing number Φ changes as *I*_*App*_ and *g*_*CaL*_ are varied. **(B)** Time series associated with the markers presented in panel A, corresponding to, from top to bottom, control conditions with relatively low *I*_*App*_, nifedipine administration with relatively low *I*_*App*_, control conditions with relatively high *I*_*App*_, and nifedipine administration with relatively high *I*_*App*_.

We once again use heatmaps and two-parameter bifurcation curves to show how changes in parameters, in this case (*I*_*App*_, *g*_*CaL*_), yield variations in the firing number Φ (Figs 5A and 6A) from the baseline condition (represented using an horizontal black dashed line). Analogous to the iberiotoxin case, the administration of nifedipine corresponds to a vertical jump from the maximum value of L-type Ca^2+^ conductance (control case), 23 (19) nS for the complex spiker (spiker) interneuron model, towards a value of 0 nS (nifedipine-treated conditions). Figs 5B and 6B show sample trajectories depicting how nifedipine alters the electrical activity in the complex spiker and spiker models, consistently producing regular action potential firing at the expense of more complex patterns. The effect of nifedipine in the model is consistent with Fig 10A in [12].

In the model of a complex spiker, with default *g*_*CaL*_ = 23 nS and low *I*_*App*_, Φ is small because the complex spiker exhibits pseudo-plateau bursting (Fig 5A). The two Φ<1 regions (grey) at the control level of *g*_*CaL*_ (for *I*_*App*_ between 50 and 150 pA or for *I*_*App*_ > 300 pA) are highly sensitive to small changes in the L-type Ca^2+^ conductance for the chosen parameters, and in both regions regular spiking activity takes over after small reductions in *g*_*CaL*_, exemplified by the simulations of nifedipine administration at *I*_*App*_ equal to 100 or 350 pA (Fig 5B).

Increasing the maximal conductance of *g*_*CaL*_ has instead an opposite effect. In fact, the region of regular spiking activity at baseline *g*_*CaL*_ disappears in response to a small increase in *g*_*CaL*_, while bursting and complex spiking regions persist and expand as *g*_*CaL*_ increases. In agreement with the results presented for the (*I*_*App*_, *g*_*BK*_) heatmap, for *I*_*App*_ between 50 and 150 pA, as *g*_*CaL*_ increases, the depolarized potentials arising during bursts stabilize and lengthen. When *g*_*CaL*_ is near 35 nS, this effect yields Φ=1; that is, the activity pattern in this part of parameter space becomes plateau bursting, such that each cycle features a long active phase with no SAOs superimposed.

Fig 6A presents analogous results for a spiker interneuron. The heatmap is predominantly white (Φ=1); as observed for the complex spiker, the complex spiking activities that arise for large *I*_*App*_ are completely abolished after a small drop in *g*_*CaL*_. Fig 6B shows trajectories depicting how the neuron behaves as the *g*_*CaL*_ conductance is changed between control and nifedipine-treated conditions for $I_{App}\in \{100,500\}$ pA, including the loss of complex spiking for *I*_*App*_ = 500 pA. In the spiker model, a small increase in the maximal conductance of the L-type Ca^2+^ channel maintains complex spiking for large *I*_*App*_ and leads to the generation of bursting phenomena for low values of *I*_*App*_. As for the complex spiker scenario, when *g*_*CaL*_ is close to 35 nS and *I*_*App*_ is between 50 and 150 pA, the model exhibits both pseudo-plateau and plateau (one LAO and no SAO) bursting and the heatmap features Φ close to or equal to 1.

L-type Ca^2+^ channels activate at a lower voltage compared to the P/Q-type Ca^2+^ channels. They depolarize the cell membrane potential as a result of Ca^2+^ influx. When they are blocked through inhibitors such as nifedipine, the depolarizing forces on the cell membrane are weaker. It is therefore, at first glance, surprising that the cell exhibiting regular spiking becomes more active when inward L-type Ca^2+^ currents are blocked (Fig 6B, upper two traces) [12]. We use our model to explain this behavior as follows. First, the resulting reduction of depolarization yields lower action potential amplitude and consequently less activation of delayed-rectifier K^+^ currents. Second, in the presence of L-type Ca^2+^ channel inhibitors there is less Ca^2+^ influx into the cell, and as a result fewer SK channels activate. The overall reduction in outward K^+^ current causes less afterhypolarization after each spike and hence allows faster regular spiking (Fig 6B, top two traces). The switch from complex spiking or pseudo-plateau bursting to regular spiking when L-type channels are blocked (or, vice versa, the switch from spiking to complex spiking and bursting when *g*_*CaL*_ is increased) is more easily explained. The reduction in depolarizing Ca^2+^ current prevents prolonged epochs of elevated membrane potential and thus promotes regular spiking in which these are absent (Fig 5B; Fig 6B bottom two traces).

For the spiker, the (*I*_*App*_, *g*_*CaL*_)-continuations of the SNP_1_, TR_1_, and PD_1_ are analogous to those shown in Figs 3A and 4A. The SNP_1_ and the TR_1_ continuations feature a cusp of SNP or TR (orange and purple circle, respectively). The TR_1_ curve, and the PD_1_ continuation in the spiker phenotype, terminate at purple and green squares, respectively, where they collide with SNPs. For the region to the left of the purple square, the SNP_1_ curve delineates the boundaries between spiking and bursting. Instead, the TR_1_ curve to the right of the purple square up to the purple circle follows the transition boundary between spiking and complex spiking only approximately, demonstrating the persistence of bistability in response to increases of *g*_*CaL*_.

### A reduced model captures CWC dynamics

The CWC model that we have discussed so far was constructed to include the key currents characterized in past experiments. Although it produces a rich repertoire of biologically relevant dynamics, its high dimensionality complicates analysis of the mechanisms involved. Hence, we next sought to produce a reduced model that maintained much of the dynamic richness of the full one, including the experimentally observed transitions with nifedipine or iberiotoxin administration.

**Fig 7:**
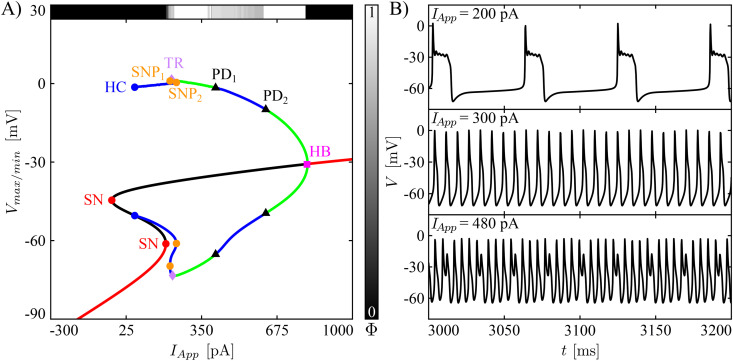
Bifurcation diagram and example voltage time series for the reduced model. **(A)** Bifurcation diagram of the reduced model (23) with respect to bifurcation parameter *I*_*App*_. The red/black continuous lines are stable/unstable fixed points, whereas the green/blue lines are the maximum and minimum voltages of stable/unstable periodic orbits. Other symbols are as in Fig 2. The horizontal bar on top of the BD shows how Φ changes as the *I*_*App*_ varies, whereas the vertical bar illustrates the colormap associated with the Φ values. **(B)** Time series of the reduced model when simulated with *I*_*App*_ equal to 200, 300 and 480 pA.

In the following, the term “full model” refers to the eleven-dimensional ODE model presented in system (1) and analyzed in the previous sections. The term “reduced model” indicates the reduction of the full model to a six-dimensional model presented in system (23), obtained through the process illustrated in section Model reduction. In brief, the reduced model was obtained from the full model by removing those currents (*I*_*NaF*_, *I*_*CaT*_, *I*_*HCN*_) that we found to be relatively small throughout the dynamic regimes of the full model. Moreover, since the KCa current was found to operate near saturation, we removed the Ca^2+^ dependency from *I*_*KCa*_. Finally, all passive currents were combined into a single leak current. These changes were found to have a relatively weak effect on the model’s spiking and complex spiking behavior. The reduced model can produce the three types of activity seen in the full model – bursting, spiking, and complex spiking (Fig 7B) – although with a shift in the *I*_*App*_ values for which these occur and a slight narrowing of the range of *I*_*App*_ for which the model exhibits bursting, compared to the full model. Moreover, because of the removal of the Ca^2+^ dynamics and the simplification of using a passive KCa current, the reduced model cannot differentiate between spiker and complex spiker cells.

In qualitative terms, the *I*_*App*_-BD for the reduced model (Fig 7A) is mostly quite similar to that of the full model in the complex spiker regime (Fig 2A). One exception is that the branch of stable equilibria (red part of the S-shaped curve) located at hyperpolarized voltages for *I*_*App*_ values below 196 pA no longer loses stability through a HB and rather maintains stability all the way up to a fold point (SN at approximately *V* = −60 mV). Consequently, a larger *I*_*App*_ is needed to elict electrical activity. Further, the range of *I*_*App*_ values for which the reduced model exhibits bursting (Fig 7B, top) is narrower than for the full model; bursting occurs from the right fold point SN to the torus bifurcation (TR), where a stable family of periodic orbits, corresponding to regular spiking (Fig 7B, middle), is born. Also, the TR_1_ and TR_2_ of Fig 2A turn into PDs (PD_1_ and PD_2_), with PD_2_ shifted to a lower value of *I*_*App*_, which implies that the reduced model exhibits complex spiking in a narrower interval of *I*_*App*_ compared with the full model. Likely, these PDs are related to PD_1_ in Fig 2B.

### Averaging theory explains CWC dynamics at different levels of blockade of L-type Ca^2+^ and BK channels

Both the full and reduced models that we have presented feature complicated timescale structures that contribute to the details of the activity patterns that they produce, with different variables evolving on at least three disparate timescales. We observed, however, that we did not need a full dissection of the model dynamics in terms of all three timescales to understand the mechanisms involved in the reduced model dynamics that we consider. That is, within the model’s three-timescale structure, the inactivation variables of the L-type Ca^2+^ (*h*_*CaL*_) and the BK-type channels (*h*_*BK*_) act as superslow variables, evolving significantly more slowly than the other model variables. We found that we could extract fundamental insights regarding the dynamic transitions induced by varying the L-type Ca^2+^ and BK channel conductances by applying averaging theory to derive averaged dynamics for these superslow quantities, without needing to consider whether the other model variables were explicitly fast or slow.

Specifically, averaging theory is used to understand how the superslow variables change when the slow and fast components of a three-timescale system evolve along a family of attracting periodic orbits of the slow-fast subsystem. For fixed values of *I*_*App*_ and other model parameters, the regions in the (*h*_*CaL*_, *h*_*BK*_) parameter plane where the theory can be applied are therefore restricted to the sets of (*h*_*CaL*_, *h*_*BK*_) where system (24) exhibits stable periodic orbits. Accordingly, we first evaluated the bifurcation structure, the attractors, and the repellers of the slow-fast subsystem with the superslow variables *h*_*CaL*_ and *h*_*BK*_ treated as parameters. These analyses required the computation of 1P- and 2P-BDs. As described in section Averaging Theory, averaging theory is applied where stable periodic orbits arise by finding a numerical approximation of the *h*_*CaL*_ and *h*_*BK*_ superslow nullclines ($NC_{h_{CaL}}$ and $NC_{h_{BK}}$) with an approach based on a periodic boundary value problem (pBVP).

**Fig 8:**
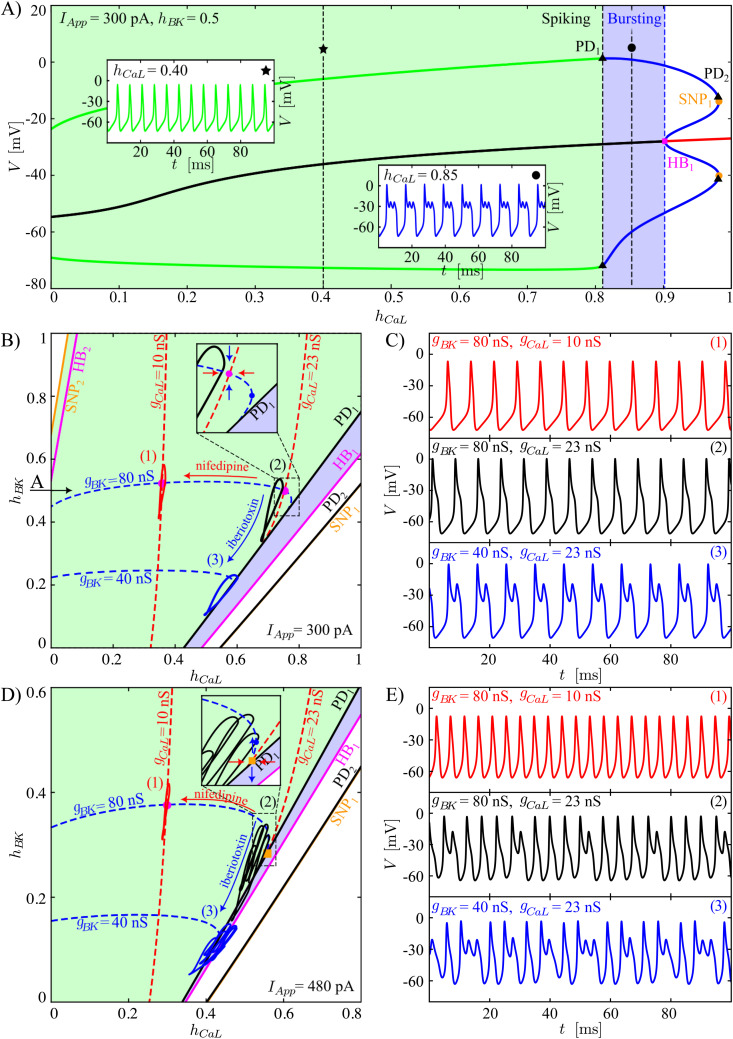
Bifurcation diagrams and time series associated with averaging theory. **(A)** 1P-BD of the slow-fast system (24) computed using *h*_*CaL*_ as a bifurcation parameter while fixing *h*_*BK*_ = 0.5 and *I*_*App*_ = 300 pA. The green and blue shaded regions correspond to intervals of *h*_*CaL*_ where the slow-fast subsystem exhibits regular spiking and bursting, respectively. The insets show, respectively, spiking and bursting solutions of the slow-fast subsystem for *h*_*CaL*_ = 0.4 (green trace, indicated by the star), respectively, *h*_*CaL*_ = 0.85 (blue trace, indicated by the dot). (B) 2P-BD of the slow-fast system (24) computed using *h*_*CaL*_ and *h*_*BK*_ as bifurcation parameters for *I*_*App*_ = 300 pA. The horizontal, black arrow at *h*_*BK*_ = 0.5 indicates the cut through this diagram corresponding to the 1P-BD in panel (A), generated with *h*_*BK*_ = 0.5. Bifurcation curves are labeled as in (A), but with additional HB_2_ and SNP_2_ curves in the upper left that are not present in (A). The red and blue dashed curves are the *h*_*CaL*_ and *h*_*BK*_ averaged nullclines, corresponding to values (*h*_*CaL*_, *h*_*BK*_) where the drift of *h*_*CaL*_ and *h*_*BK*_, respectively, averaged around one cycle of the stable slow-fast periodic orbit, is zero. The arrows in the main panel indicate how these averaged nullclines shift with reductions in corresponding conductances representing drug application. The labels (1), (2), (3) are associated to the nearby closed curves illustrating the reduced model behaviour projected onto the considered phase plane and correspond to the voltage traces shown in panel (C). The inset is a zoom around the equilibrium point under baseline conditions. The blue dot represents the fold of the *h*_*BK*_ superslow average nullcline, while the red and blue arrows indicate the direction of the superslow average variation close to the stable equilibrium point of the superslow averaged dynamics (magenta dot) along the *h*_*CaL*_ and *h*_*BK*_ axes, respectively. (C) Reduced-model time series for *I*_*App*_ = 300 pA for each simulation labelled (1)-(3) in panel (B). (D, E) Analogous to panels (B) and (C) but for *I*_*App*_ = 480 pA. The orange square indicates a superslow averaged saddle equilibrium point.

Fig 8A shows a 1P-BD of the slow-fast subsystem computed using *h*_*CaL*_ as the bifurcation parameter, while fixing *h*_*BK*_ and *I*_*App*_ at 0.5 and 300 pA, respectively. In this diagram, starting from low values of *h*_*CaL*_, the BD features a branch of stable periodic orbits (green) and a branch of unstable equilibria (black), hence the slow-fast subsystem exhibits regular spiking (as shown in the simulation for *h*_*CaL*_ = 0.40 presented in the inset of the plot). As *h*_*CaL*_ increases, the branch of stable limit cycles destabilizes through a PD (PD_1_), and the branch of equilibria subsequently gains stability through a subcritical HB (HB_1_). For *h*_*CaL*_ in between these two bifurcations, which occur at 0.810 and 0.902 (blue shaded region), the bifurcation diagram does not show any attractor. Simulation of the slow-fast subsystem showed that in this parameter interval, the slow-fast subsystem produces bursting (as shown in the simulation for *h*_*CaL*_ = 0.85 presented in the inset) with a number of SAOs that increases as *h*_*CaL*_ moves closer to HB_1_. A similar type of bifurcation structure has been observed in our recent work on layer V cortical neurons [28]. In that case, the mechanism for the generation of SAOs in the slow-fast subsystem was related to the existence of a folded node, but we leave the analysis of the mechanism underlying the SAOs in the CWC models for future investigations. Finally, for values of *h*_*CaL*_ between HB_1_ and its maximal value 1, the slow-fast subsystem converges towards the depolarized stable equilibrium.

By following each of the bifurcation points identified in the 1P-BD based on *h*_*CaL*_ under variations of *h*_*BK*_, we next produced a 2P-BD. In the 2P-BD, the region of slow-fast periodic dynamics (Figs 8B and 8D, green shaded regions) is bounded in the direction of increasing *h*_*CaL*_ by a curve of period doubling bifurcations (Figs 8B and 8D, PD_1_ curves). We found that for *h*_*BK*_ sufficiently positive, this region is bounded below by an SNP curve (Fig 8B, SNP_2_), although this curve was not evident in the 1P-BD, which corresponds to the horizontal arrow at *h*_*BK*_ = 0.5 in Fig 8B, for which this curve does not lie in the physiological regime, *h*_*CaL*_ ≥ 0, depicted in the 1P-BD. Within the region of slow-fast periodic dynamics, we numerically constructed the superslow averaged nullclines (dashed blue curve for the *h*_*BK*_ nullcline and dashed red curve for the *h*_*CaL*_ nullcline). Each was computed both for a baseline condition and for a pharmacological manipulation corresponding to a reduction in the conductance of the relevant current. Inhibition of the L-type Ca^2+^ channels led to a leftward shift of the *h*_*CaL*_ superslow averaged nullcline, while inhibition of the BK channels led to a downward shift of the *h*_*BK*_ superslow averaged nullcline.

Any intersection point of the superslow averaged nullclines represents an equilibrium point of the superslow dynamics, corresponding to net zero drift of the superslow variables over one full cycle of the underlying slow-fast periodic oscillation. The stability of the superslow equilibrium point was assessed by evaluating the superslow drift in its neighbourhood (arrows in the insets of Fig 8BD). For baseline conductances (*g*_*BK*_ and *g*_*CaL*_ equal to 80 and 23 nS, respectively), the superslow equilibrium point is stable (Fig 8B, magenta dot). Thus, we predict that the overall reduced model will produce regular spiking, which is exactly what we observe in the phase space (closed black curve in Fig 8B, (2)) and as a time series (Fig 8C, (2)). When a moderate dose of iberiotoxin is administered (hence *g*_*BK*_ is changed from 80 to 40 nS), $NC_{h_{BK}}$ shifts downward, while $NC_{h_{CaL}}$ remains unchanged. In this case, as $NC_{h_{BK}}$ moves, the superslow stable equilibrium point slides along $NC_{h_{CaL}}$, and disappears through the line PD_1_. The disappearance of the superslow equilibrium point provides the dynamic mechanism underlying the transition from spiking to bursting in the reduced model (Figs 8B and 8C, (3), blue curves), with a corresponding quantative estimate for the *g*_*BK*_ value at which this event will occur. Alternatively, partial inhibition of L-type Ca^2+^ channels shifts $NC_{h_{CaL}}$ leftward, leaving $NC_{h_{BK}}$ unaltered. Therefore, in this case, the super-slow equilibrium point moves leftward along the averaged *h*_*BK*_ nullcline $NC_{h_{BK}}$ and maintains its stability, hence regular spiking persists (Figs 8B and 8C, (1), red curves).

We repeated the same analysis for a case where the baseline dynamics is complex spiking rather than regular spiking by taking *I*_*App*_ = 480 pA (Figs 8D and 8E, (2), black)). Here, the superslow averaged equilibrium point in the control condition exists but is unstable (orange square) and close to both the fold of $NC_{h_{BK}}$ and to the line PD_1_. The instability of the equilibrium point predicts the existence of a complex form of dynamics in the reduced model. As in the previous case, reduction of the maximal BK channel conductance *g*_*BK*_ eliminates the equilibrium point (Figs 8D and 8E, (3), blue). A new effect here is that reduction of *g*_*CaL*_ shifts the equilibrium point along $NC_{h_{BK}}$ and causes it to gain stability, switching the model from complex to regular spiking (Figs 8D and 8E, (1), red).

## Discussion

This work presents a biophysical model of cartwheel interneurons, integrating information from and capturing the transitions observed in a variety of single-cell experimental studies [11,12,17,27,29,30]. In this paper, we focus on: (1) providing a computational demonstration of the difference between two classes of cartwheel interneurons, the spiker and the complex spiker, which confirms the experimentally motivated distinction of CWC types suggested in [12] and suggests how the two classes’ dynamics are related, and (2) elucidating the behavior of the CWCs in response to administration of iberiotoxin and nifedipine, which are pharmacological BK and L-type Ca^2+^ channel blockers [12], based on full or partial reduction of the corresponding conductances.

The model was investigated through numerical simulation and production of heatmaps (Figs 3–6) illustrating how the firing number [24], which reflects some of the morphological properties associated with a time series, changes under variation of model parameters *g*_*CaL*_, *g*_*BK*_, and *I*_*App*_ along with two-parameter bifurcation curves. Unlike biological experiments, our numerical approach easily allows us to consider gradual changes in parameter values over regions of parameter space. The heatmaps highlight the relative fragility versus robustness of model dynamics as these parameters were varied, depending on the baseline regime of model activity.

Specifically, our model predicts that complex spikers can exhibit regular spiking but that this disappears when *g*_*BK*_ is slightly decreased (in response to low iberiotoxin concentrations). The exact degree of sensitivity to iberiotoxin may, of course, vary across biological interneurons; our model predicts that regular spiking will be more robust in those with higher levels of baseline BK channel conductances. Regular spikers are also predicted to exhibit complex spiking under iberiotoxin administration, but are predicted to require a significantly larger reduction in BK conductance before this change in activity occurs. Importantly, our modeling suggests that the larger baseline *g*_*KV*_ and *g*_*CaL*_ and smaller baseline *g*_*NaP*_ and *g*_*KCa*_ in the complex spiker case corresponds to a qualitative difference in bifurcation structure as *I*_*App*_ is varied, relative to the regular spiker regime. We predict that this difference will manifest in which form of dynamics will be first observed in the transition from quiescence to ongoing spiking as *I*_*App*_ is increased. In our simulations, both model types show a rapid loss of complex phenomena (i.e., complex spiking and bursting) with simulated nifedipine application. This result agrees with the observations in [12], where the administration of nifedipine significantly diminished the likelihood of generation of complex spikes.

To explain the effects of simulated pharmacological manipulations, we simplified our full model to a six-dimensional reduced model that retained the responses to current variations observed in complex spiker CWCs and preserved the dynamic transitions observed with iberotoxin and nifedipine treatment. Specifically, the reduced model exhibits bursting, spiking, and complex spiking as observed in a complex spiker CWC in response to increasing current stimulation. In terms of their dynamics, the variables in the reduced model segregate into three distinct timescale classes. To study the effects of iberiotoxin and nifedipine, however, we find that it suffices to group the fast and slow variables together and to consider the dynamics of *h*_*BK*_ and *h*_*CaL*_, which evolve on the slowest (superslow) timescale, averaged over oscillations arising in the slow-fast subsystem. The observation that a full dissection of variables into their separate timecales classes is sometimes not necessary for understanding system dynamics fits with other recent works [26,28,31] on three-timescale systems. Nonetheless, it is somewhat surprising that in the regular spiking regime in the reduced model, the slow-fast nature of the ongoing oscillations is not clearly evident in the appearance of the time series (Fig 8B simulations (1),(2) and Fig 8D (1)).

Within the context of the reduced model, we used averaging theory to numerically estimate the superslow nullclines in the region of (*h*_*BK*_, *h*_*CaL*_) where the slow-fast subsystem of the model, comprising its other variables, exhibits periodic oscillations. We then studied how the existence and stability of the superslow equilibrium point, where these nullclines intersect, changes under variation of *g*_*BK*_ and *g*_*CaL*_ for two different values of *I*_*App*_ corresponding to two different baseline configurations of relevant dynamical structures. The results show that regular spiking patterns correspond to a stable superslow equilibrium. Under parameter variations, this equilibrium can destabilize by passage through the fold of the *h*_*BK*_ averaged nullcline (Fig 8D, from (1) to (2)) and can be lost when the intersection point of the nullclines reaches a curve of slow-fast system PD bifurcations (Fig 8BD, from (2) to (3)). The latter contrasts with homoclinic, saddle-node of periodic orbit, and torus bifurcations that bound regions of fast subsystem oscillations in other models (e.g., [26,32]) and matches what we see in the full system bifurcation diagram for the reduced model computed with respect to the parameter *I*_*App*_ (Fig 7A). Interestingly, the destabilization of the averaged superslow equilibrium point gives rise to a pattern in which complex spikes alternate with regular spikes (e.g., in a 3–1 ratio in Fig 8E simulation (2)), whereas the loss of the equilibrium produces periodic complex spikes in one parameter regime (Fig 8BC simulation (3)) and a seemingly-chaotic mixture of regular and complex spikes in another (Fig 8DE simulation (3)). Our analysis does not predict or explain the nature of these patterns; however, period doubling has previously been linked to spike adding in bursting dynamics [33–36], and further analysis of these patterns, presumably relating to all three timescales present in the system, remains open for future work.

Finally, we note that, consistent with the results presented in the heatmaps, averaging theory predicts which types of electrical activity will be promoted in response to an increase in *g*_*BK*_ or *g*_*CaL*_, which is a new prediction that has not been tested experimentally. Specifically, if *g*_*BK*_ increases, then in our model, the *h*_*BK*_ nullcline will be shifted upward, promoting the stabilization of the super-slow equilibrium point and predicting the existence of a regular spiking solution. Instead, if *g*_*CaL*_ increases, the *h*_*CaL*_ nullcline will be shifted rightward, moving the super-slow equilibrium point along the $NC_{h_{BK}}$, inducing a loss of stability and the emergence of complex spiking and bursting solutions.

This work provides a useful tool to address more complicated biological and mathematical questions in future research. First, in [16], it was hypothesized that complex spiking plays a crucial role in network communication at microcircuit level. Our model provides a computational platform that can be used to study the importance of complex spiking at the small network level, for example to improve the understanding of the interactions between CWCs and fusiform neurons. Second, although our averaging analysis did exploit the multiple-timescale nature of our model, we did not pursue a more complete analysis of its timescale structure. Future studies should focus on a more detailed mathematical analysis of the various forms of complex spiking and bursting that our model produces, including the mechanisms underlying the SAOs and the transitions between bursting, spiking, and complex spiking in response to variation of *I*_*App*_, *g*_*CaL*_, and *g*_*BK*_.

The heatmaps and 2P-BDs in Figs 3–6 provide predictions regarding the transitions of the CWC behaviour under inhibition/enhancement of BK and L-type Ca^2+^ currents for different levels of *I*_*App*_. The dynamic clamp technique [37,38] may be a valuable tool for testing these predictions, and hence the accuracy of our model, experimentally. Specifically, after iberiotoxin adminstration, a patched CWC interneuron could be stimulated with a computed BK current for comparison to our simulation outcomes.

To conclude, in this paper, we presented a novel biophysical ODE-based model of CWCs able to generate bursting, spiking, and complex spiking phenomena. Our analysis highlights a possible distinction between CWCs labeled as complex spikers versus spikers in previous experiments and predicts how effects of variations in an applied stimulation current and changes of BK and L-type Ca^2+^ channel conductances will modulate the electrical activity of these neurons. This work represents a first analytical step towards elucidating the mechanisms mediating the generation of complex phenomena in CWCs and how plasticity affecting L-type Ca^2+^ and BK channel conductances might influence their electrical activity and hence auditory function and dysfunction.

## Materials and methods

### Model

We constructed a biophysical, conductance-based, whole-cell model for individual cartwheel interneurons (CWCs) using information derived from a variety of experiments. The model equations take the form


$$\begin{array}{cc} C_{m}\dot{V} & =-I_{K}-I_{Ca}-I_{Na}-I_{HCN}-I_{L}+I_{App},\\ \dot{p} & =[p_{\infty }(V)-p]/\tau_{p}(V),\\ \dot{Ca} & =-f\;(\alpha I_{Ca}+kCa), \end{array}$$ (1)

where *C*_*m*_ is the membrane capacitance, *V* is the membrane potential, *Ca* represents the calcium concentration in the intracellular compartment and, finally, *p* denotes a generic gating variable of an ion channel. Specifically, $p\in \{m_{KV},m_{KDR},m_{BK},h_{BK},$ $h_{CaL},m_{CaT},h_{CaT},h_{NaF},m_{HCN}\}$, where the notation *m*_*X*_ indicates activation variables, while *h*_*X*_ are inactivation variables. The right-hand side of the *p* equation includes the functions $p_{\infty }(V)$ and $\tau_{p}(V)$, which are the steady-state and the time-scale functions, respectively. Calcium dynamics is modeled using a single compartment capturing the intracellular calcium concentration with a linear elimination rate *k*, where *f* is the ratio of free-to-total cytosolic calcium and α is a coefficient converting from current to molar flux. The terms *I*_*K*_, *I*_*Ca*_, and *I*_*Na*_ are the overall K^+^-, Ca^2+^- and Na^+^ currents, defined as


$$\begin{array}{cc} I_{K} & =I_{KDR}+I_{KV}+I_{BK}+I_{KCa}+I_{KATP}, \end{array}$$ (2)

$$\begin{array}{cc} I_{Ca} & =I_{CaPQ}+I_{CaL}+I_{CaT}, \end{array}$$ (3)

$$\begin{array}{cc} I_{Na} & =I_{NaF}+I_{NaP}+I_{NaL}. \end{array}$$ (4)

Their extended definitions are presented below and justified in the following subsections.

First, the total K^+^ current *I*_*K*_ is composed of delayed-rectifier (*I*_*KDR*_), voltage-gated (*I*_*KV*_), big-conductance (*I*_*BK*_), Ca^2+^-gated (*I*_*KCa*_) and ATP-dependent K^+^ (*I*_*KATP*_) currents. These take the forms


$$\begin{array}{cc} I_{KDR} & =g_{KDR}\;m_{KDR}\;(V-E_{K}), \end{array}$$ (5)

$$\begin{array}{cc} I_{KV} & =g_{KV}\;m_{KV}^{4}\;(V-E_{K}), \end{array}$$ (6)

$$\begin{array}{cc} I_{BK} & =g_{BK}\;m_{BK}\;h_{BK}\;(V-E_{K}), \end{array}$$ (7)

$$\begin{array}{cc} I_{KCa} & =g_{KCa}\;m_{KCa,\infty }(Ca)\;(V-E_{K}), \end{array}$$ (8)

$$\begin{array}{cc} I_{KATP} & =g_{KATP}\;(V-E_{K}). \end{array}$$ (9)

Influx of Ca^2+^ into the intracellular compartment is due to P/Q-, L-, and T-type Ca^2+^ currents (*I*_*CaPQ*_, *I*_*CaL*_, *I*_*CaT*_, respectively). The definitions of these currents are


$$\begin{array}{cc} I_{CaPQ} & =g_{CaPQ}\;m_{CaPQ,\infty }(V)\;(V-E_{Ca}), \end{array}$$ (10)

$$\begin{array}{cc} I_{CaL} & =g_{CaL}\;m_{CaL,\infty }(V)\;h_{CaL}\;(V-E_{Ca}), \end{array}$$ (11)

$$I_{CaT}=g_{CaT}\;m_{CaT}\;h_{CaT}\;(V-E_{Ca}).$$ (12)

The Na^+^ currents in the model are a fast/transient type (*I*_*NaF*_), a persistent type (*I*_*NaP*_), and a Na^+^ leak current (*I*_*NaL*_), which are described by


$$\begin{array}{cc} I_{NaF} & =g_{NaF}\;m_{NaF,\infty }^{3}(V)\;h_{NaF}\;(V-E_{Na}), \end{array}$$ (13)

$$\begin{array}{cc} I_{NaP} & =g_{NaP}\;m_{NaP,\infty }^{3}(V)\;(V-E_{Na}), \end{array}$$ (14)

$$\begin{array}{cc} I_{NaL} & =g_{NaL}\;(V-E_{NaL}). \end{array}$$ (15)

Finally, we considered three additional currents: the hyperpolarization-activated cyclic nucleotide-gated current (*I*_*HCN*_) and a general leakage current (*I*_*L*_), given by


$$\begin{array}{cc} I_{HCN} & =g_{HCN}\;m_{HCN}\;(V-E_{HCN}), \end{array}$$ (16)

$$\begin{array}{cc} I_{L} & =g_{L}\;(V-E_{L}), \end{array}$$ (17)

and an externally applied current denoted by the constant *I*_*App*_.

All the steady-state activation and inactivation functions implemented in the model, unless otherwise stated, take the form


$$p_{\infty }(V)=\frac{1}{1+\exp[-(V-v_{p})/s_{p}]}.$$ (18)

However, the steady-state activation of the Ca^2+^-gated K^+^ channel is modeled as


$$m_{KCa,\infty }(Ca)=\frac{Ca^{n}}{Ca^{n}+k_{Ca}^{n}}$$ (19)

and the inactivation variable of the BK channel is modeled using a simplified formulation of the model presented in [39], where the inactivation level is controlled by the calcium concentration in the nanodomain below the mouth of P/Q-type Ca^2+^-channels. Hence,


$$h_{BK,\infty }(V)=\frac{\gamma }{\delta \;K\;m_{CaPQ,\infty }(V)\;|V-E_{Ca}|+\gamma }.$$ (20)

The general expression for the timescale function in (1) is


$$\tau_{p}(V)=T_{p0}+\frac{T_{p}}{\exp[-(V-v_{p1})/s_{p1}]+\exp[-(V-v_{p2})/s_{p2}]}.$$ (21)

Note that the timescale function can be chosen to be modelled as voltage-independent by setting *T*_*p*_ = 0. Parameters related to the dynamics of the various gating variables are listed in Table 1. Table 2 presents the different Nernst potentials and the parameters associated with calcium dynamics, and Table 3 summarizes the values of the conductances used for a spiker and for a complex spiker CWC.

**Table 1:** Parameters used to define the kinetic properties of the ion channels involved in the model. The *STANDARD* section presents values of the parameters of the channels obeying equations (18) and (21). Those activation and inactivation variables with a voltage-independent timescale have *T*_*p*_ = 0 and *T*_*p*0_ different from zero. Those activation and inactivation variables that are assumed to be in steady-state have a ‘-’ in the columns from *T*_*p*0_ to *s*_*p*2_. The *NON STANDARD* section provides values of the remaining parameters characterizing activation of the KCa channels through equation (19) and inactivation of the BK channels via equation (20).

STANDARD										
CH.	*m*/*h*	*v*_*p*_	*s*_*p*_	*T*_*p*0_	*T*_*p*_	*v*_*p*1_	*v*_*p*2_	*s*_*p*1_	*s*_*p*2_	REF.
		[mV]	[mV]	[ms]	[ms]	[mV]	[mV]	[mV]	[mV]	
KDR	*m*	-11	8	0	13	-100	50	-39	65	[27]
KV	*m*	-55	15	2	0	–	–	–	–	[18]
BK	*m*	-22	8	2	0	–	–	–	–	[17]
	*h*	–	–	5	0	–	–	–	–	
CaPQ	*m*	-22	8	–	–	–	–	–	–	[17]
CaL	*m*	-38	6	–	–	–	–	–	–	[40]
	*h*	-42	-4	20	0	–	–	–	–	
CaT	*m*	-40	9	0.4	7	-120	-40	240	-10	[41]
	*h*	-93	-10	8	500	-93	-93	10	-10	
NaF	*m*	-35	7	–	–	–	–	–	–	[42]
	*h*	-77	-9	0	13	-70	-54	-15	44	
NaP	*m*	-50	15	–	–	–	–	–	–	[42]
HCN	*m*	-90	-15	30	0	–	–	–	–	[11]
**NON STANDARD**										
**CH.**	***m*/*h***	***n***	***k*_*Ca*_**	** δ **	***K***	** γ **	**–**	**–**	**–**	REF.
		[]	[μM]	[$\frac{1}{\mu M\;ms}$]	[$\frac{\mu \text{M}}{\text{mV}}$]	[${\text{ms}}^{-1}$]	[]	[]	[]	
KCa	*m*	2	0.4	–	–	–	–	–	–	[43,44]
BK	*h*	–	–	0.0025	0.67	0.002	–	–	–	[39]

**Table 2:** This table shows the parameters of the model associated with the Nernst potentials (*E*_*Ca*_, *E*_*K*_, *E*_*L*_, *E*_*Na*_, *E*_*HCN*_ and *E*_*NaL*_), the membrane capacitance (*C*_*m*_), and the dynamics of the intracellular calcium compartment (*f*, *k* and α).

GENERAL								
*E*_*Ca*_	60	[mV]	*E*_*Na*_	50	[mV]	*f*	0.05	[]
*E*_*K*_	-85	[mV]	*E*_*HCN*_	-20	[mV]	*k*	0.3	[ms^-1^]
*E*_*L*_	-40	[mV]	*E*_*NaL*_	0	[mV]	α	0.003	[μM pA^-1^ ms^-1^]
*C*_*m*_	10	[pF]						

**Table 3:** This table shows the conductances of a spiker (S)/complex spiker (CS) CWC model.

CONDUCTANCES [nS]								
*g_X_*	CS	S	*g_X_*	CS	S	*g_X_*	CS	S
*g_KDR_*	100	100	*g_CaPQ_*	5	5	*g_NaP_*	31	33
*g_KV_*	32	29	*g_CaL_*	23	19	*g_NaF_*	100	100
*g_BK_*	80	80	*g_CaT_*	5	5	*g_NaF_*	1	1
*g_BK_*	6	6	*g_KCa_*	10	11	*g_JCN_*	5	5
*g_L_*	0.1	0.1						

### Potassium channels

Potassium ions move across the cellular membrane through different channels, e.g., Ca^2+^-gated K^+^ channels [12,17,29], Kv2 channels [27] and ATP-dependent K^+^ channels [11]. Experiments [12,17,29] have established the existence of big- and small-conductance K^+^ channels (BK, SK, respectively). In the model, we implement only two Ca^2+^-gated K^+^ channels, the BK and the KCa ones. The latter accounts for both the small- and the intermediate-conductance K^+^ channel contributions.

Our model assumes that BK channels activate shortly after the formation of microdomains below open P/Q-type Ca^2+^ channels, which implies that the steady state functions of BK and P/Q-type Ca^2+^ currents are proportional [39]. The inactivation of the BK channels is modeled using a simplified formulation of the conductance-based model presented in [39]. The parameters γ, *K*, and δ are derived directly from [39]. We adjusted the steady-state activation functions for these channels and the BK maximal conductance to replicate the voltage-clamp experiments presented in Fig 4Aii in [17] and to capture the dynamical transition between the regime of simple and complex spiking presented in Fig 7A in [12]. The KCa channels are modeled through a Ca^2+^-dependent steady-state activation function, as presented in equation (19), derived from [43]. The value of *k*_*Ca*_ is increased to 0.4 μM, which is the average of the values used in two previous models [43,44]. The maximal conductance of the KCa current is constrained to match that of previously-studied apamine-sensitive currents [17,29]. The properties of Kv2 channels (i.e., one fundamental component of the delayed-rectifier K^+^ class) and their dynamics, specifically the *v*_*p*_ and *s*_*p*_ parameters of the steady-state activation function and the voltage-dependent time constant, were fit to the normalized conductance data obtained through voltage-clamp experiments presented in Fig 3 in [27]. The term *T*_*p*_ presented in this paper is scaled by a temperature factor, changing from 30 to 13 ms, which is specific for the Kv2 channels, to account for the temperature difference between the experiments in [27] and the simulations presented here. The maximal conductance of the KDR channel was set to capture the dynamical transition between simple/complex spiking to a silent depolarized state after the administration of guangxitoxin (a Kv2 inhibitor). The ATP-dependent K^+^ current was modelled as a passive current. The maximal conductance *g*_*KATP*_ was estimated to match the tolbutamide-sensitive currents detected in [11]. Specifically, in [11], the available measures distinguish between silent- and active-cartwheel when no current is applied. We aim to build a model of a silent interneuron; hence, we replicated the results associated with a silent cell presented in Fig 9G in [11]. During the construction of the model, we observed the necessity of a hyperpolarizing current to promote the activation of the small amplitude oscillations observed in complex spiking and bursting conditions. Therefore, we assumed the existence of an additional generic, voltage-gated K^+^ current (KV) modelled as in [18]. The maximal conductance *g*_*KV*_ was manually tuned to allow the model to exhibit bursting, spiking, and complex spiking dynamics.

### Sodium channels

Na^+^ is one of the fundamental components contributing to the generation of the electrical activity of the CWCs [12,30]. The total Na^+^ current in these neurons is composed of TTX-sensitive and TTX-insensitive components. The former is due to persistent- and transient-type Na^+^ channels, whereas the latter reflects Na^+^ leak channels (NaLCN). The dynamics of the persistent and transient-type Na^+^ channels are taken from [42]; the maximal conductance of the persistent-Na^+^ channel *g*_*NaP*_ is constrained by the voltage-clamp data presented in Fig 4C in [11], while that of the transient-type Na^+^ channels is manually chosen to be 3 times bigger than *g*_*NaP*_. The TTX-insensitive current, *I*_*NaL*_, is modelled as a leakage current, with a maximal conductance chosen to be of the same order of magnitude as *g*_*KATP*_.

### Calcium channels

Kim et al. [12] found L-, T- and P/Q- types Ca^2+^ currents in CWCs. Another study made on a non-specific population of neurons of the DCN [45] found the same results. The dynamics of the P/Q-type Ca^2+^ currents in our model are adapted to meet the voltage-clamp experiments in [17]. The dynamics of L-type Ca^2+^ currents, on the other hand, are taken from [40] and adjusted. Specifically, we changed the half-width activation of the steady-state function (*v*_*p*_) to −38 mV in our model, and we considered the inactivation timescale of the L-type calcium current to be constant and set at 20 ms. The maximal conductances of the L-, T-, and P/Q-type channels were chosen to replicate the experimental transitions presented in [12], that is, to promote the transition from simple to complex spiking in response to agatoxin (a P/Q-type Ca^2+^ channel blocker) as suggested in Fig 9A and Fig 9C in [12], and the non-effect of mibefradil (a T-type Ca^2+^ channel blocker) when the neuron is under a current stimulus, as presented in [[12], Fig 11B]. Finally, the T-type Ca^2+^ currents were taken from [42,41], which presented a model of cerebellar Purkinje interneurons.

### Hyperpolarization-activated cyclic nucleotide-gated channel

Another type of channel identified in CWCs in [11,12] is the HCN channel. All the parameters associated with the model of this channel class were adjusted to match the voltage-clamp experiments presented in Fig 6E in [11]. The adopted HCN channel model has the structure initially presented in [46], and recently used in [28], which is justified by the similar I/V relationships seen in [11] and [46].

### Numerical methods

XPPAUT [47] was the primary tool used to solve the model system of ODEs, using the qualitative Runge-Kutta method, which is designed to preserve solution properties (e.g., positivity of gating variables), with a time step of 0.01 ms. The absolute and relative tolerances used to solve the ODE system were set to 1e-6. For the heatmap creation, we identified spikes based on crossings of a Poincaré section defined with respect to the variable *V* over a 3-second, uninterrupted simulation. MATLAB [48] was used to control the routines for the creation of heatmaps running on a computer cluster, for offline data analysis, and for figure creation. Finally, Inkscape [49] was used for image editing.

### Firing number

We computed a morphological index, the firing number [24], based on the properties of complex spiking and pseudo-plateau bursting dynamics, to quantify model activity. The voltage traces of these activity patterns include both full action potentials, or large amplitude oscillations (LAOs), and spikelets, or small amplitude oscillations (SAOs). Thanks to the existence of SAOs, these patterns resemble mixed-mode oscillations. For this reason, they can be summarized through their signature $L_{1}^{s_{1}}L_{2}^{s_{2}}...$ [50]. The signature of a time series can be periodic or aperiodic. In both cases, $L_{i},s_{i}\in \mathbb{N}$ indicate the number of LAOs and SAOs, respectively, in a cycle. Therefore, for any simulated time series comprising *n* cycles, we can define $L=\sum_{i=1}^{n}L_{i}$ and $s=\sum_{i=1}^{n}s_{i}$, and calculate the firing number [24] as follows:


$$\Phi =\left\{ \begin{array}{cc} 0 & \text{if}\;L\;=\;\text{s }=\;0,\}\\ L/(L+s) & otherwise. \end{array}\right.$$ (22)

By construction, Φ∈[0,1]. Specifically, Φ=1 when the time series is comprised entirely of LAOs so that *s* = 0. Alternatively, Φ=0 when *L* = 0, hence when the neuron exhibits SAOs only or is silent. For Φ values between 0 and 1, the time series features SAOs on top of a plateau. In this latter case, we cannot distinguish between pseudo-plateau bursting and complex spiking. However, this index can help us to discriminate between these complex patterns and regular spiking activity.

### Heatmaps

The heatmaps are constructed to go beyond the experimental results based on all-or-none channel blockage by systematically investigating how the model behaves for intermediate levels of reduction of certain transmembrane current(s) in a specific range of applied current. Partial current reductions can be simulated by changing the maximal conductance associated with a single or a set of ion channels.

We changed *I*_*App*_ and either *g*_*BK*_ or *g*_*CaL*_ over a predefined grid and, with these parameters given by each grid point, calculated the firing number Φ from the model activity. The maps were created via the following three steps:

*Step 1 - Update ICs*: For each value of the conductance (*g*_*BK*_ or *g*_*CaL*_) in the grid, we define a new initial condition (IC) by simulating the neuron for 2 s, starting from a common, fixed baseline IC. In this pre-simulation, a constant *I*_*App*_ drives the neuron to a stable steady state, which is then used as the IC for the next step. The value of the applied current taken to represent the rest condition for the model is defined to be, for the given conductance value, the largest integer multiple of −10 pA below the current $I_{App,HB_{1}}$ corresponding to the HB_1_ on the hyperpolarized branch of equilibria (cf. Fig 2). That is, since the location of the HB changes when *g*_*BK*_ or *g*_*CaL*_ changes, so does the value of the pre-stimulation current representing the rest condition.

*Step 2 - Model simulation*: For each value of *I*_*App*_ and *g*_*BK*_ (or *g*_*CaL*_) in the grid, we simulate the response of the neuron for 3 s, starting from the IC found as the steady-state in Step 1. We discard an initial transient of 1 s of the simulation, and save only the local maxima and minima of the remaining part of the voltage time series.

*Step 3 - Index computation*: For any combination of *I*_*App*_ and *g*_*BK*_ (or *g*_*CaL*_) in the grid, the associated series of peaks is used to calculate the firing number Φ for this grid point. More details regarding the analyses of the sequences of peaks and valleys used to calculate the index Φ are given in the Peak analysis section.

### Peak analysis

This algorithm analyzes the sequence of maxima and minima associated with a time series. It distinguishes between spiking and complex phenomena, e.g., pseudo-plateau bursting and complex spiking, and it extracts the signature of the time series to calculate the firing number Φ. The algorithm comprises the following steps:

*Step 1 - Cleaning*: For values of *I*_*App*_ close to HB_1_ in the full model, the trajectory of the system exhibits SAOs occuring around a hyperpolarized voltage. However, these subthreshold fluctuations are not of interest for calculating the firing number, which accounts only for the SAOs occuring around a depolarized plateau. For this reason, we omit the subthreshold maxima and minima from the calculation of the firing number.

*Step 2 - Identification*: From the cleaned data, a series of peaks/valleys is extracted.

*Step 3 - Modality*: A test is performed to determine whether the *V*-coordinates of the valleys form a multimodal distribution. If so, the time series is associated with a complex activity pattern, and we proceed with step 4. Otherwise, the time series can be classified as regular spiking, hence Φ=1 and the algorithm terminates.

*Step 4 - Classification*: The sequence of peaks is classified through an adaptive threshold calculated using the Otsu algorithm [51].

*Step 5 - Cropping*: The classified vector of peaks is cropped at its beginning/end so that the sequence starts/ends with a peak associated with a LAO/SAO.

*Step 6 - Calculation*: The series of maxima is converted into the sequence $L_{1}^{s_{1}}...L_{n}^{s_{n}}$. The sample is then used to compute Φ through the formulas mentioned in section Firing number.

## Model reduction

The model reduction process retains only some features of the full model. The procedure considers a model of a complex spiker and aims to produce a new model that (1) captures the transitions observed experimentally in response to administration of iberiotoxin and nifedipine and (2) maintains the transitions between bursting, spiking, and complex spiking under variation of *I*_*App*_.

The reduced model takes the form


$$\begin{array}{cc} C_{m}\dot{V} & =-{\tilde{I}}_{L}-I_{CaPQ}-I_{CaL}-I_{NaP}-I_{KDR}-I_{KV}-I_{BK}+I_{App},\\ \dot{p} & =[p_{\infty }(V)-p]\tau_{p}^{-1}, \end{array}$$ (23)

with $p\in \{m_{KDR},m_{KV},m_{BK},h_{BK},h_{CaL}\}$. The values of some model parameters are those presented in Tables 1, 2 and 3, while others were modified. The form of the reduced model and the choice to alter certain parameters were based on the following reasoning:

First, we observed that the heatmaps computed by varying *I*_*App*_ and *g*_*HCN*_, *g*_*CaT*_, or *g*_*NaF*_ were not strongly sensitive to conductance variations. For this reason, despite the biological evidence for the existence of these currents in CWCs, we decided to remove these currents from the model by setting their conductances to 0 nS. This step slightly alters the *I*_*App*_ associated with the bifurcations detected in the *I*_*App*_-BD presented in Fig 2. However, with this simplification, the model still satisfies conditions (1) and (2).

In the spiking and the complex spiking conditions, the relationship between the calcium concentration and the L-type Ca^2+^ inactivation variable in the full model was linear. Moreover, for high *I*_*App*_, the KCa current was always operating at saturation, effectively acting as a leakage current. For this reason, we decided first to incorporate the KCa current into the K(ATP) current through a modification of the conductance of the latter from 6 to 17 nS. Then, we removed the calcium dynamics.

To follow the ideas presented in [52], we fixed the value of the KDR timescale at $\tau_{m_{KDR}}=1.3$ ms, thereby obtaining a constant-τ model.

For the sake of compactness, we condense the leakage-type currents, *I*_*KCa*_, *I*_*NaL*_, *I*_*KATP*_, and *I*_*L*_, into a new leakage current (${\tilde{I}}_{L}$) whose conductance (${\tilde{g}}_{L}$) and reversal potential (${\tilde{E}}_{L}$) are equal to 18.1 nS and -80 mV, respectively.

These simplifications altered the model rheobase and narrowed the interval of *I*_*App*_ where the reduced model of complex spiker CWCs exhibits bursting. Moreover, the bifurcation diagram of the reduced model does not have an HB in the hyperpolarized branch of stable equilibria. This bifurcation likely disappears in a fold-Hopf bifurcation as it encounters the SN as part of the model reduction.

The reduced model obtained through these steps cannot differentiate between two different types of interneurons, spikers and complex spikers. Due to its low-dimensionality, however, it provides a useful setting in which to apply mathematical theories to explain the transitions between different activity regimes arising at different levels of inhibition of BK and L-type Ca^2+^ channels.

### Averaging theory

Averaging theory can be used in dynamical systems with multiple timescales to understand how the slowest variables will drift along an attracting family of periodic orbits of the subsystem composed of the faster variables [25,26,32]. In this work, it is employed to discuss the fragility and robustness of the regular spiking presented in Figs 3 and 5.

To apply this theory, we classified the timescales of the reduced model dynamical variables using a standard nondimensionalization procedure for *V* and inspection of the explicit timescales $\tau_{p}$ for the other variables, see equation (21) and Table 1. The transmembrane potential *V* evolves with a rate that is approximately 0.1 ms; the gating variables *m*_*KDR*_, *m*_*KV*_ and *m*_*BK*_ vary with rates between 1 and 2 ms; the rate of *h*_*BK*_ is 5 ms; and finally, the rate of *h*_*CaL*_ is 20 ms. In this scenario, we classify the variable *V* as fast, *m*_*KDR*_, *m*_*KV*_ and *m*_*BK*_ as slow and finally *h*_*BK*_ and *h*_*CaL*_ as superslow. Thus, we can define the slow-fast subsystem of equation (23) as follows:


$$\begin{array}{cc} C_{m}\dot{V} & =-{\tilde{I}}_{L}-I_{CaPQ}-I_{CaL}-I_{NaP}-I_{KDR}-I_{KV}-I_{BK}+I_{App},\\ \dot{p} & =[p_{\infty }(V)-p]\tau_{p}^{-1}, \end{array}$$ (24)

with $p\in \{m_{KDR},\;m_{KV},\;m_{BK}\}$. In the system presented in equation (24), the slowest variables of the reduced model, *h*_*BK*_ and *h*_*CaL*_, are considered as parameters.

The averaging theory is applied by numerically reconstructing the superslow averaged nullclines and studying the stability of any superslow equilibrium points where these nullclines intersect. In the following, the procedure to compute the *h*_*BK*_ superslow nullcline ($NC_{h_{BK}}$) is presented. A similar approach has been adopted for the calculation of the *h*_*CaL*_ superslow average nullcline ($NC_{h_{CaL}}$). We indicate with γ a stable limit cycle existing for a given combination of the parameters *h*_*CaL*_ and *h*_*BK*_ in the slow-fast subsystem presented in equation (24). We define the superslow average variation along γ as


$$\langle \dot{h_{BK}}\rangle =\frac{1}{T_{\gamma }}\langle h_{BK}\rangle^{*}:=\frac{1}{T_{\gamma }}\int_{0}^{T_{\gamma }}g_{h_{BK}}(V_{\gamma }(t),h_{BK})dt.$$ (25)

The function $g_{h_{BK}}(\cdot )$ corresponds to the right-hand side of the *h*_*BK*_ differential equation in the reduced model. $V_{\gamma }(t)$ indicates the temporal evolution of the *V*-coordinate of γ, while $T_{\gamma }$ is the period of γ. On the right hand side of equation (25), *h*_*BK*_ is required to take values where a limit cycle γ exists. The quantity $\langle \dot{h_{BK}}\rangle$ represents the average variation along γ of the superslow variable *h*_*BK*_. The *h*_*BK*_ superslow average nullcline is


$$NC_{h_{BK}}=\{(h_{CaL},\;h_{BK})\in {\mathbb{R}}^{2}\;|\;\langle \dot{h_{BK}}\rangle =0\}.$$ (26)

To compute this set of points, we adapted an approach based on continuation of solutions to a pBVP presented in [32]. We rescale *t* in equation (24) by $T_{\gamma }$, hence $\tilde{t}=t/T_{\gamma }$, and we extend the system with three additional dynamical variables, namely $\langle h_{BK}\rangle$, *h*_*BK*_, and $T_{\gamma }$, as follows:


$$\begin{array}{cc} C_{m}V^{\prime } & =-T_{\gamma }({\tilde{I}}_{L}+I_{CaPQ}+I_{CaL}+I_{NaP}+I_{KDR}+I_{KV}+I_{BK}-I_{App}),\\ p^{\prime } & =T_{\gamma }[p_{\infty }(V)-p]\tau_{p}^{-1},\\ {\langle h_{BK}\rangle }^{\prime } & =T_{\gamma }[h_{BK,\infty }(V)-h_{BK}]\tau_{h_{BK}}^{-1},\\ h_{BK}^{\prime } & =0,\\ T_{\gamma }^{\prime } & =0, \end{array}$$ (27)

where the prime symbol denotes differentiation with respect to the new time variable $\tilde{t}$. The last two equations in (27) act as free parameters in the numerical continuation. The limit cycle γ is still a solution of the extended system in equation (27) but now, with respect to the new temporal variable $\tilde{t}$, the period is unitary. The system in equation (27) is solved using the periodic, boundary, and initial conditions


$$\begin{array}{cc} V(0) & =v_{0},\\ V(1) & =v_{0},\\ p(0) & =p(1),\\ \langle h_{BK}\rangle (0) & =0,\\ \langle h_{BK}\rangle (1) & =\langle h_{BK}\rangle^{*}. \end{array}$$ (28)

The pBVP (27), (28) can be solved only if the dynamical variables and the parameter *v*_0_ are properly initialized. Specifically, given γ, we must select an arbitrary point $(V,p)\in {\mathbb{R}}^{4}$ so that (V,p)∈γ, and then use the *V*-coordinate to initialize *v*_0_, while using the remaining coordinates to initialize the periodic conditions set for the dynamical variables *p*. We suggest to select a point (*V*,*p*) along γ so that the value of *V* is close to the average of the variable’s excursion range. Choosing a value close to the extrema can interrupt the continuation too early. Finally, $\langle h_{BK}\rangle^{*}$ is the value computed through the integral in equation (25) along γ. The construction of $NC_{h_{BK}}$ is completed through the following four steps:

*Step 1 - 1P-BD:* The one-parameter bifurcation diagram (1P-BD) of the slow-fast subsystem in (24) is computed using *h*_*BK*_ as the bifurcation parameter by fixing *h*_*CaL*_ to 0. We performed this step using XPPAUT [47], which we also used to calculate an approximation of all the stable limit cycles in the branch of detected stable periodics (NPR is set to 1).

*Step 2 - Initialization:* The resulting BD is loaded in MATLAB with XPPLORE [53] to compute $\langle h_{BK}\rangle^{*}$ for every stable limit cycle saved from the generation of the 1P-BD. After this step, the limit cycle with the lowest $\left|\langle h_{BK}\rangle^{*}\right|$ is selected and the values of *h*_*CaL*_, *h*_*BK*_, $T_{\gamma }$ and $\langle h_{BK}\rangle^{*}$ are extracted to initialize the *ode* file containing an implementation of system (27) together with the conditions in (28).

*Step 3 - Continuation:* First, AUTO in the XPPAUT package is used to find a γ value where $\langle h_{BK}\rangle (1)$ is zero through a numerical continuation along the $\langle h_{BK}\rangle^{*}$ parameter. Second, the solution to the pBVP is continued along *h*_*CaL*_. This last step calculates $NC_{h_{BK}}$.

*Step 4 - Cropping:* In the last step, the outcome of the previous numerical continuation is loaded in MATLAB through XPPLORE and the curve is cropped in the region of the parameter space (*h*_*CaL*_, *h*_*BK*_) where the slow-fast subsystem in (24) admits stable limit cycles.

The same method is iterated to calculate the super-slow nullclines $NC_{h_{BK}}$ and $NC_{h_{CaL}}$ for different values of *I*_*App*_. Finally, the super-slow equilibrium point is obtained as the intersection between $NC_{h_{BK}}$ and $NC_{h_{CaL}}$, estimated numerically. Its stability is investigated through visual inspection of the flow around the point based on numerical simulations.
